# The Joint Research Centre-European Network of Cancer Registries Quality Check Software (JRC-ENCR QCS)

**DOI:** 10.3389/fonc.2023.1250195

**Published:** 2023-10-26

**Authors:** Francesco Giusti, Carmen Martos, Stefano Adriani, Manuela Flego, Raquel Negrão Carvalho, Manola Bettio, Enrico Ben

**Affiliations:** ^1^ European Commission, Joint Research Centre (JRC), Ispra, Italy; ^2^ Belgian Cancer Registry, Brussels, Belgium; ^3^ Foundation for the Promotion of Health and Biomedical Research in the Valencian Region (FISABIO), Valencia, Spain

**Keywords:** cancer registry, validation, harmonisation, data quality, software, Europe

## Abstract

The core activity of population-based cancer registries (PBCRs) is to gather information from all new cancer cases in a defined geographic area, in order to measure the magnitude of cancer burden and to provide a basis for cancer research. The Joint Research Centre-European Network of Cancer Registries Quality Check Software (JRC-ENCR QCS) is a Java standalone desktop application, under development since 2015, created to support PBCRs in the validation of the collected data. The JRC-ENCR QCS performs internal consistency checks on the cancer registry dataset, to detect impossible or unlikely codes or combination of codes, and is thereby an important tool to support the validation efforts by registries and improve data quality and European-wide harmonisation. The software package also includes the JRC CSV Data layout converter, a complementary tool for transforming PBCR incidence files into a format compatible with the JRC-ENCR QCS. This paper gives an overview of the JRC-ENCR QCS, describing the role of the software in processing data files submitted by PBCRs contributing to the European Cancer Information System (ECIS) as well as its functionalities. The development of the JRC-ENCR QCS is an evolving process, with regular updates implementing new and revised European and International recommendations and classifications.

## 1 Introduction

Population-based cancer registries (PBCRs) systematically collect data from all new reportable cancer cases occurring in a defined geographic area (1). In Europe, PBCRs are organised within the European Network of Cancer Registries (ENCR), established in 1989 in the framework of the Europe Against Cancer Programme of the European Commission. The ENCR is a professional, non-profit society dedicated to promoting collaboration between PBCRs, defining data collection standards and providing training to PBCR personnel. It aims to strengthen the basis for monitoring cancer burden in the EU and the rest of Europe, through the provision of regular and timely information from European PBCRs.

The European Cancer Information System (ECIS) was developed by the European Commission’s Joint Research Centre (JRC) in collaboration with the Directorate-General for Health and Food Safety (DG SANTE), following the 2009 “*Communication on Action Against Cancer: European Partnership*” (2). Launched in 2018, the ECIS provides indicators (incidence, mortality and survival) to quantify cancer burden across Europe thanks to the contribution of cancer registries data (3) through periodic data calls. A dedicated data submission portal (4) was developed to collect registry data files for incidence, mortality, population and life tables, submitted by ENCR registries according to a well-defined protocol (5) that details the list of variables and allowed range of values required for the calculation of cancer burden indicators and publication in ECIS.

The reliability and use of the information provided by PBCRs depend on data quality, measured through its different dimensions of comparability, completeness, validity and timeliness (6–8).

Adherence to protocol, data standardisation and internal consistency checks are the core steps of the data validation process carried out by the JRC to ensure harmonisation and comparability of European PBCRs data.

In support to this process, an ENCR expert working group (WG) published a comprehensive and standardised list of data quality checks to be adopted by European PBCRs and European projects.

The WG addressed case and variables definition, format for data collection and related internal consistency rules. The results of the initiative were ENCR-endorsed reports (9–11) which serve as guidelines for the data acquisition and further validation of PBCRs data.

This work was also the basis for the development of the JRC-ENCR Data Quality Check Software (JRC-ENCR QCS) described here, an open-access software to facilitate standardisation and validation of PBCR data (12). The aim of this paper is to give an overview of the JRC-ENCR QCS by describing its role in processing data files submitted by PBCRs contributing to the ECIS, and list its main validation routines.

## 2 Method

### 2.1 Overview of the JRC-ENCR QCS

The JRC-ENCR QCS has been designed as a standalone desktop application that can run locally by PBCRs, without the need of internet connection.

Processing cancer data locally is a common precautionary practice to protect sensitive patient health data from external access, thus allowing PBCRs to directly check and correct their data files while avoiding the stringent General Data Protection Regulation (GDPR) rules that must be applied when sharing individual data, even after pseudonymisation. Incidence files are considered sensitive even if pseudonymised, as they contain patient’s sensitive data, such as date of birth, date of cancer diagnosis, sex, geographical location, therefore, in order to be compliant with the GDPR regulation, incidence data must be handled with the proper precautions (13).

The software was developed using an almost pure Java design pattern in which strictly necessary libraries are used either to reduce the number of dependencies or to avoid vendor’s lock-ins.

The JRC-ENCR QCS has a flexible architecture and can perform checks on different data collection protocols, such as the 2015 and 2022 ECIS protocols (5, 14).

The standard execution of the software is the Graphical User Interface (GUI) mode, which opens the GUI window and waits for actions from the user. Alternatively, the JRC-ENCR QCS may be run in the command-line mode, validating the dataset passed as an argument.

Regardless of the execution mode, output reports are produced in the output subfolder within the directory where the software is installed.

The JRC-ENCR QCS has been initially developed for the Windows operating system, requiring Java (Windows 8 and above). Starting from JRC-ENCR QCS version 1.7 it can also run on MacOS and Linux operating systems.

Since the first release, the software has been upgraded and improved based on the JRC Technical reports “*A common data quality check procedure for European cancer registries*” (9–11), on the new ENCR recommendations (15), on the experience acquired in data validation for ECIS and on the feedback received from the PBCR users.

### 2.2 The dataset

The inputs required by the JRC-ENCR QCS are text files, with default data fields (variables) delimited by semicolon (“;”). The software configuration allows also for comma-separated variables.

The software is able to check four different data files (incidence, mortality, population and life tables), required to update all the incidence, mortality and survival indicators published in ECIS, and the calculation of different quality indicators.

The cancer incidence file contains different groups of variables:

- variables related to the patient,
- variables related to the tumour,
- variables related to the follow-up,
- variables related to stage and
- variables related to treatment.

When PBCR contribute with their incidence file to European projects, they are responsible for the prior pseudonymisation of their data. This is the case of the contribution to ECIS, which requires the upload of pseudonymised data in the JRC data submission portal.

Information about the processing of data is published at the European Commission’s Register of the Data Protection Officer (DPO) and available at the following link: https://ec.europa.eu/dpo-register/detail/DPR-EC-00417.

There is correspondence between the population and cancer incidence files with respect to registration area, period, sex, age-range and geographical reference codes. The variables included in the population file are: calendar year, sex, age, geographical area code and label, as well as number of residents. The information included in the population file should be obtained from official censuses, from intercensal/postcensal estimates provided by Vital Statistics Departments, or equivalent, or other official sources.

The mortality data corresponds to the cancer incidence file with respect to registration area, period, sex and age-range. The mortality information is obtained from the Vital Statistics Department, or equivalent, and based on certificates/death records. The file should contain the following variables: calendar year, sex, age, cause of death and number of deaths.

Life tables file must be provided by registries covering their entire period of incidence or the period in which the follow-up is available. Life tables have the same geographical and temporal reference as for the cases of the incidence file and contain the following variables: calendar year, sex, age, geographical area code and label, as well as all causes of death probability.

### 2.3 The data check process

**Figure 1:**
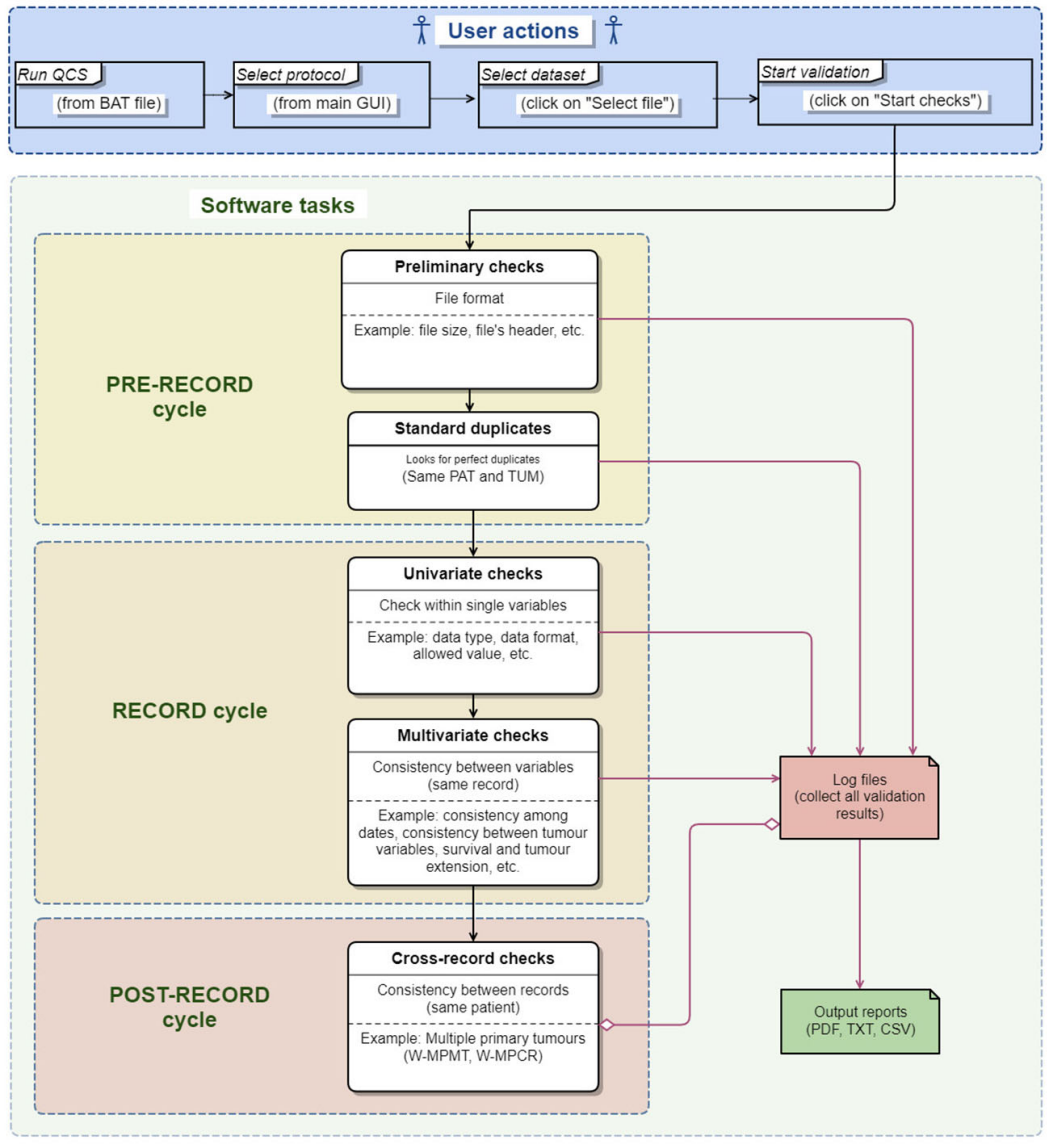
Data quality check process performed by the JRC-ENCR QCS.

Data checks are performed in consecutive cycles, each comprised of different steps (**Figure 1**). Generally, data checks fall within three main categories:

- Checks on single variables (univariate checks)
- Checks between variables of the same record (multivariate checks)
- Checks between variables of different records belonging to the same patient (perfect duplicates and multiple primary tumours checks)

Population, life tables and mortality files have a simple structure that can be validated by univariate checks only. On the other hand, incidence files are more complex, as they contain more variables (the ECIS protocol requested 56 and 39 variables for the 2015 and 2022 data calls respectively). This data can be recorded/codified in different ways, and can be more prone to errors. For this reason, in addition to the checks on single variables, the checks on the incidence file should include as well checks between variables of the same record.

Additionally, the JRC-ENCR QCS also checks for the presence of multiple primary tumours in the incidence file, which is an important validation step for the correct ascertainment of number of cancer cases. This is because the same patient can be associated to multiple tumours, recorded either at the same time or at different times, and that can represent the same primary tumour or completely independent tumours.

The time needed to perform all the checks and create the output reports depends mainly on the computer’s memory; the performance of the process increases significantly starting from 4GB of RAM. A second factor affecting the process is the number of errors/warnings. There is an approximately linear relation between the number of errors/warnings and the time spent to finalise the validation of data. For instance, with 4GB of RAM, 100,000 records and circa one error/warning per record the total time of the process is around 2 minutes, and around 20 minutes with one million records.

The minimum number of records checked by the software is one, while the maximum is related to the number of errors/warnings, at around 10 million messages (e.g. 1 million records with 10 errors/warnings per line).

### 2.4 Univariate checks

Univariate checks on single variables are the simplest checks, used to verify that the value of each variable is compliant with the required format and is within the range specified by the protocol. An example is the variable “sex” which must be codified as numeric and has four allowed values: 1=male, 2=female, 3=other or 9=unknown.

Sometimes the software has to adapt the theoretical range of values allowed by the protocol to the values that are used (and submitted) by PBCRs. This is the case of the “*pN*” variable, i.e. the pathological assessment of the regional lymph nodes in the TNM classification system (16). In 2021, the JRC-ENCR QCS expanded the set of allowed values for “*pN*” in order to include also the TNM notation “*1biv*”, coming from previous TNM editions. Such TNM notation was still in use by some PBCRs, and resulted in an excessive amount of “out of range” errors identified in the software output, shadowing the actual critical errors in the dataset.

### 2.5 Multivariate checks of the same record

Multivariate checks identify inconsistencies among values in different variables of the same record. Some checks, such as comparing topography and morphology codes, are straightforward and are performed according to a well-defined table of allowed/refused combinations of cancer morphologies and topographies.

By contrast, the consistency between age/topography/morphology, which is required because some cancer types occur almost exclusively in certain age groups, is one of the most complex checks. This is the case of retinoblastoma (tumour of young children) or prostate cancer (in older men). Therefore, some combinations of age/topography/morphology are unlikely or very rare and should produce a warning message according to the list of unlikely/rare combinations of age/topography/morphology. Additionally, several morphology codes may be related to multiple topography values, thus increasing the complexity in the checks for age/topography/morphology combinations (11). For example, in the age group 0-14 years, about 50 different morphology codes for the gonadal carcinoma type can be combined with the two topography values C56 (ovary) and C62 (testis), producing about 100 different unlikely combinations.

### 2.6 Multivariate checks between different records of the same patient

The implementation of the multivariate checks between different records of the same patient is rather complex. Firstly, not all records in the dataset need to be checked, only records that meet certain requirements and pass the univariate or multivariate checks (Sections 2.4 and 2.5) must be compared against each other. This implies that the software has to store the results of all the basic checks before deciding which records should be evaluated by the rules for the multivariate checks between different records of the same patient.

Secondly, some complex filtering criteria must be applied on the dataset, including those on the behaviour of multiple primary tumours. These are quite critical and at the same time, have been subject to several changes in the latest years due to updates of the international cancer coding rules, new ENCR recommendations for coding and reporting tumours, new requirements from the PBCRs, results of data checks, etc.

Finally, while in the dataset each patient is identified by a patient ID, records are usually not ordered by patient ID and two or more records addressing the same patient may not be in consecutive rows of the data file. The JRC-ENCR QCS is not designed to store in memory the entire dataset, which could contain millions of rows and would require a computer with a large amount of memory (the procedure for these checks is detailed in Section 2.7.2).

The software performs two checks between records of the same patient: the first identifies perfect duplicates (records with same patient and tumour IDs) in the pre-record cycle (**Figure 1**) and the second addresses Multiple Primary Tumours (MPT) (records with same patient ID and different tumour ID). The rules used for checking MPT take place in the Post-record cycle (**Figure 1**) since they are usually performed after the basic univariate and multivariate checks described in the previous sections.

This could be the case of a patient with multiple metastases originating from a single primary tumour but recorded separately at different times. The multiple primaries rule can identify multiple records of the same patient that refer to the same primary tumour, reducing the risk of counting the same tumour twice (or more). This check, and the subsequent elimination of these records from the dataset must be performed before data aggregation, and is crucial to correctly calculate the cancer indicators published on the ECIS website.

The pre-record cycle, record cycle and post-record cycle, each consisting of several steps are described in more detail in the following sections.

### 2.7 Validation workflow

#### 2.7.1 File structure validation (pre-record cycle)

In this validation phase, only preliminary rules (e.g. checking the file format, file size, file header, looking for perfect duplicates) are applied. Depending on the JRC-ENCR QCS configuration, some errors found during these checks, such those occurring when a dataset is empty or too small, can stop the execution of the program and interrupt the validation process (blocking errors).
Log files (e.g. *qcs_rule_output.csv*) collect all errors/warnings identified at any stage of the validation process and are used to produce the final output reports.

#### 2.7.2 Record cycle

If the whole dataset passes the checks of the previous validation phase, further checks are performed as described below.

All records of the input dataset are read and processed one by one, and all basic rules (univariate and multivariate checks) are applied to each record independently. This approach optimises the memory management and allows the JRC-ENCR QCS to analyse big datasets (with millions of rows) efficiently as only one record at time is kept in memory to be processed. During the record cycle, issues can be detected in each record for single variables (e.g. data format, data type, data range) and/or between variables (e.g. coherence between topography and morphology, consistency between age and tumour type).

After a record has been checked, but before moving on to the next record, a special notification is sent according to all the rules (e.g. MPT rule) applied in the next post-record cycle. This notification contains the list of errors/warnings found in the processed record, including critical errors on some core variables (e.g. date of incidence or topography) that, according to specific acceptance criteria, prevent the software from applying such rules. The acceptance criteria and the related list of critical errors are specific for each rule.

For example, if the JRC-ENCR QCS identifies an error E-MISS (value missing) on the variable *YoI* (Year of Incidence) this is considered critical and the MPT rule of the post-record cycle (described in the following section) cannot be applied to the record with this type of error. The same error on the variable “*Stage*” is not critical and the record could be accepted and processed.

All acceptable records are stored in a temporary file. For example, if two rules 1 and 2 are defined in the post-record cycle, two log files will be produced (e.g. *qcs_acceptable_by_rule_1.csv*, *qcs_acceptable_by_rule_2.csv*), each with the list of records accepted by the specific rule according to its respective criteria.

These log files usually contain fewer records than the original dataset, depending on the acceptance criteria of the corresponding rule. For example, the list of records acceptable for the MPT rule will not include records with critical errors on core variables.

At the end of the record cycle, all errors and warnings produced for each record are added to the log files created in the first validation phase: these log files will be later used to produce the final output files.

#### 2.7.3 Sorting of temporary files

The process of sorting temporary files produced in the record cycle occurs between the record and post-record cycles. Acceptable records stored in the temporary files are sorted according to some criteria, specific for each rule. For example, for the MPT rule all records are sorted by patient ID, so that consecutive records with the same patient ID can be easily analysed without scanning and saving into the computer memory the whole dataset for each patient ID.

The list of sorted acceptable records produced during this step is saved in a new temporary file. For example, file *qcs_acceptable_by_rule_1-by-PAT.csv* will include the list of records which are “acceptable” for the MPT rule and sorted by patient ID. These records are ready to be processed in the next validation cycle.

#### 2.7.4 Post-record cycle

In the post-record cycle, a set of rules which perform specific checks between records is applied to the temporary files produced in the previous cycle. All records are compared to each other according to specific criteria defined for each rule. During this process, a set of specific errors and warnings is produced.

The new MPT rules have been defined and implemented in 2022 and will be available in the next version of the JRC-ENCR QCS.

When these rules are applied, records in the temporary files are read in batches, meaning that all records having the same patient ID are loaded and handled at the same time. At this point, some specific filtering criteria (e.g. on the tumour’s behaviour) can be applied to exclude some records from the batch. For example, a record with topography code C50 (breast) and behaviour 0 (benign) will be ignored, while all tumours with behaviour 3 (malignant) will be processed, in line with the current call for data protocol (5).

For each batch of records with the same patient ID, some equivalence criteria on the morphology and topography codes are then applied to check if two tumours can be considered the same primary tumour according to the rules for checking solid MPTs defined in the quality check report “*A common data quality check procedure for European cancer registries*” (11).

### 2.8 Producing output reports

When all validation steps have been completed, the log files, which include all errors and warnings collected during the validation process, are used to produce the final output.

Each log file is sorted with respect to the original ordering. This is necessary to print all errors and warnings for each record exactly in the same order of the original dataset.

### 2.9 Outcome of the validation process

All the checks performed by the software are following the International Classification of Diseases for Oncology, Third Edition (ICD-O-3) topography and morphology codes (17).

After all the checks described above (univariate, multivariate and between records checks) have been executed, the JRC-ENCR QCS produces some output reports listing all messages collected during the validation process. Output messages can be warnings (i.e. the record should be reviewed by the PBCR) or errors (the record cannot be accepted as it is and should be corrected by the PBCR).

In some rare cases, the software can produce also critical messages, meaning that something went fatally wrong. This could be the case of a “broken rule” (e.g. the user tampered with the configuration files and removed a resource used by a specific check) or it could be the case of a dataset that cannot be read correctly (e.g. wrong number of columns, wrong separator between variables).

Two types of messages are printed in the output reports: W-YYYY (warning code) and E-YYYY (error code), where the code YYYY identifies the specific type of message.

Below some examples:

- W-AGMT: Unlikely Age + tumour type
- W-BDMO: Morphology too specific taking into account the basis of diagnosis
- W-MOTO: Morphology + Topography not valid
- E-FORM: Format error
- E-MISS: Value missing
- E-OUTR: Value out of range

The list of all error and warning codes used by the JRC-ENCR QCS is available in the header of the PDF and TXT output reports.

### 2.10 The output reports

Each validation run generates three output files in PDF, TXT and CSV format. The PDF file contains a summary of the execution process (date and time, name of the processed file, number of rows, total number of errors and warnings) and the detailed list of all errors and warnings detected for each record of the input data file. The TXT output file has the same content of the PDF, but in text format.

The CSV file contains all errors and warnings in a format easily readable by automated procedures. This file can be used by users to load the results of the validation process in a database, or to perform detailed statistics and analyses. This format can be particularly useful if the input file generates a large number of errors.

### 2.11 Protocol and application configurations

Starting from version 2.1 the software can perform the validation of the input data set according to different versions of the protocol, 2014 and 2020, corresponding respectively to the protocols for the JRC-ENCR calls for data in 2015 and 2022. These documents detail the guidelines for submitting to the JRC four types of data files: incidence, mortality, population and life tables. The software can run in 10 different modalities, according to the specific data call protocol. These modalities are referred to as “protocols”:

- Incidence 2014: Incidence protocol 2014 (56 variables)
- Incidence 2020: Incidence protocol 2020 (39 variables)
- Mortality 2014: Mortality protocol 2014 (5 variables)
- Mortality 2020 - Age Unit: Mortality protocol 2020 “Age Unit” (5 variables)
- Mortality 2020 - Age Range: Mortality protocol 2020 “Age Range” (5 variables)
- Population 2014: Population protocol 2014 (4 variables)
- Population 2020 - Age Unit: Population protocol 2020 “Age Unit” (6 variables)
- Population 2020 - Age Range: Population protocol 2020 “Age Range” (6 variables)
- Life Table 2014: Life Table protocol 2014 (4 variables)
- Life Table 2020: Life Table protocol 2020 (6 variables)

A summary of the number and type of files needed to configure the JRC-ENCR QCS application is specified below:

- About 10 configuration files for the general configuration
- About 70 configuration files for defining the list of variables and the allowed range of values
- About 10 configuration files for defining the protocols and the list of rules
- About 40 configuration files for defining the internal logic of each validation rule

## 3 Results

### 3.1 The role of the JRC-ENCR QCS in the data processing workflow

As a first step, the PBCR should extract data from its database to create the incidence file, following the requirements of the ENCR call for data protocol (4). This step is not always straightforward, since data might be extracted with a slightly different format or structure, or the PBCR might use a different coding for the variables than the one specified in the protocol. Due to the large number of data submissions to JRC-ENCR deviating from the format requested in the data call protocol, either because of time or technical constraints from the PBCR side, the CSV Data Layout Converter has been developed as an auxiliary tool to facilitate the preparation of the incidence file by the PBCR before running the software.

In the second step, the PBCR should run the software on the four different files to be submitted (incidence, population, mortality, life table). All records having some issues will produce errors and/or warnings in the output files, allowing the PBCR to verify and possibly correct the issues before data submission.

When the files are ready, the PBCR submits them through the secure JRC Data Submission Portal.

After data is submitted by the PBCR, JRC re-runs the JRC CSV Data layout converter on incidence data to check the adherence of the incidence file to the format required by the ENCR protocol and verifies that the file can be validated by the JRC-ENCR QCS. The preliminary format check is carried out by the JRC for all data files submitted by the PBCR. The results of this first check are communicated to the PBCR via the JRC Data Submission Portal with a preliminary format check report.

In case of critical format errors, it might not be possible for the JRC to correct them and run the software. In this case the PBCR will be asked to correct the format and submit again the dataset to JRC.

If the files are received in the correct format, the JRC will check them running the software and will prepare an internal consistency report. A summary of the submitted data, with the number of records for each type of error and warning, is included in this report. Issues raised by the JRC-ENCR QCS for which there is a clear solution (e.g. a prostate cancer case with topography C61 instead of C61.9) are included in the internal consistency report only to inform the PBCR on the proposed solution (e.g. topography is changed to C61.9).

If needed, the PBCR will fix the issues reported by the JRC and will re-submit the updated file through the JRC portal.

In order to apply the international rules for multiple primary tumours (18, 19), the JRC is defining and developing a new MPT algorithm for automatic selection of multiple primary tumours according to current international rules for these tumours. The MPT algorithm will be applied on incidence data only after the cleaning process.

The new advanced features that will be available with the JRC-ENCR QCS version 2.1, will offer several improvements:

- Full implementation of all the 10 protocols
- Better usage of the computer’s memory during validation
- Better definition of multiple primary criteria (updated to the latest guidelines)
- More precise identification and classification of MPTs
- More precise filtering criteria for MPT with behaviour < 3 (non-malignant behaviours)
- A richer validation feedback to the user (both in the GUI and in the output reports)
- Updates of all univariate and multivariate checks according to the latest guidelines

Some more advanced improvements are planned for version 2.2, such as:

- Possibility to exclude duplicate records from the dataset
- Possibility to merge duplicate records into a unique one

The final steps performed at JRC are data aggregation, calculation of quality indicators such as incidence rates, and publication of the results in the ECIS web application.

**Figure 2:**
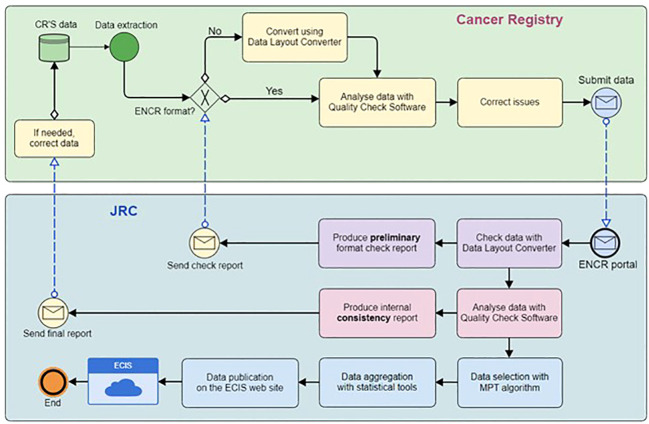
ECIS cancer burden data flow from submission to publication.

**Figure 2** illustrates the validation workflow of an incidence file. Parallel considerations could be done for the other types of files (mortality, population, and life table).

### 3.2 History and roadmap of the JRC-ENCR QCS development

The development of the software occurred in subsequent versions, following incorporation of feedback from several PBCRs. In 2015 the first version of JRC-ENCR QCS (1.4) was released for testing by European PBCRs. This version contained all basic checks (univariate and multivariate), but not the MPT checks. Some versions (1.5 and 1.6) were released in 2016 informally (at the JRC level, and to few pilot PBCRs) to test and verify the accuracy of the MPT check.

An updated version 1.7 of the software was officially released in November 2016: this was the first public available version which included the MPT check, and the possibility to run also on MacOS and Linux operating systems.

This release was followed by version 1.8 in December 2018, which included new features and many corrections, thanks to the feedback received by PBCRs. The main new features of JRC-ENCR QCS 1.8 were the creation of a separate file reporting on MPTs and the update of morphology families used by the MPT checks according to the 2011 update of ICD-O-3.

The latest release of the JRC-ENCR QCS is version 2.0, based on the guidelines of the ENCR data call protocol 2022 and on the experience gathered in validating more than 35 million cases submitted in the first JRC-ENCR 2015 data call from around 150 PBCRs based in 35 European countries.

Version 2.0 introduced TNM (Tumour/Nodes/Metastasis) (16) consistency checks and further updates of ICD-O-3 morphology codes. Moreover, the new 2.0 version introduced a completely new architecture, which moved all protocol data from the source code to some configuration files. This new approach made it possible to update the logic of the majority of checks without the need to release a new version of the software, but simply updating the configuration files.

PBCRs can download the latest version of the JRC-ENCR QCS toolkit, which include the *CSV Data Layout Converter* tool and the latest version of the JRC-ENCR QCS, from the ENCR website (20).

The JRC in collaboration with the ENCR has been organising several training sessions to familiarise PBCRs staff with the software (21).

### 3.3 Using the JRC-ENCR QCS

**Figure 3:**
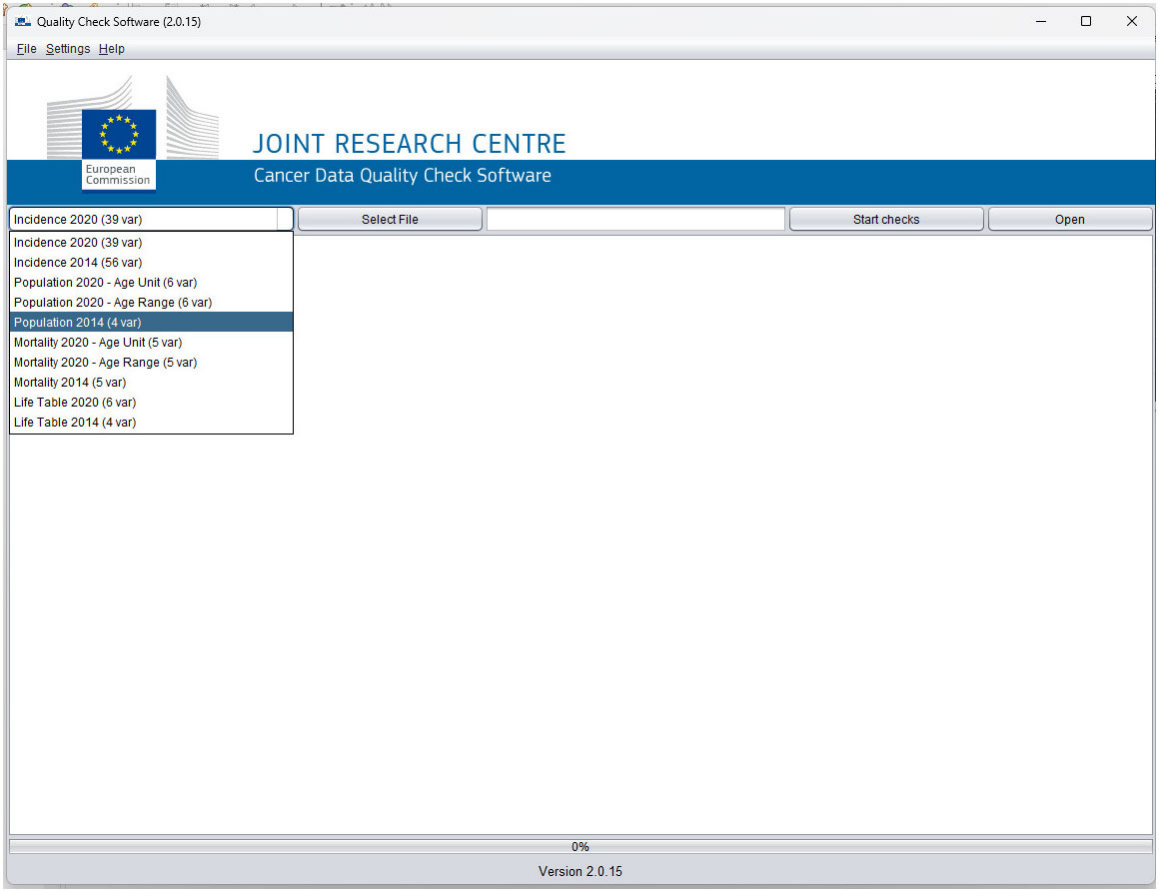
The user interface of the JRC-ENCR QCS (Version 2.0.15 example).

The software use is rather straightforward; after launching it the user interface window is opened (**Figure 3**).

The user can choose the type of file and the data call protocol from the drop-down menu. By selecting “*Incidence 2014 (56 variables)*”, the validation checks are performed according to the 2015 data call protocol, whereas “*Incidence 2020 (39 variables)*” corresponds to the rules of the 2022 data call.

The “*Select File*” button allows browsing and selecting the file to be checked, and the “*Start checks*” button starts the validation process. While the software is running, the number of the checked records appears in the display text box. Once the validation is ended, the output window displays a short report about the completed process, while the “*Open*” button allows accessing the output folder containing all the output report files.

Similarly, mortality, population and life table files can be checked by selecting the corresponding type of file from the drop-down menu.

**Figure 4:**
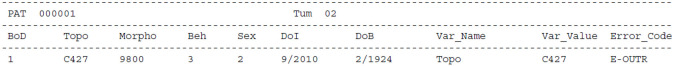
Error for out of range value for topography (E-OUTR). Topography C427 does not exist in the ICD-O-3 classification.

**Figure 5:**
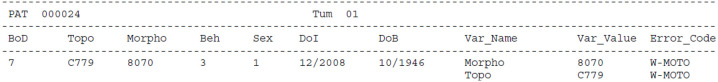
Warning for morphology and topography combinations (W-MOTO). The combination of topography=C779 (Lymph node, NOS) and morphology=8070 (squamous cell carcinoma, NOS) is probably a metastasis and topography should be coded as C809 for the unknown primary site.

**Figure 6:**
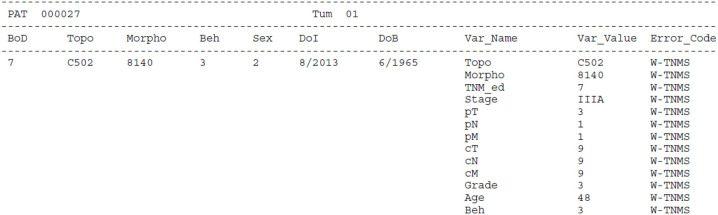
Warning for inconsistency between TNM and stage (W-TNMS). This case is a breast carcinoma with pT=3, pN=1, pM=1 and Stage=IIIA. This combination is not consistent with the TNM classification (7th edition); this means that either pM is actually 0, or stage is equal to IV.

**Figure 7:**
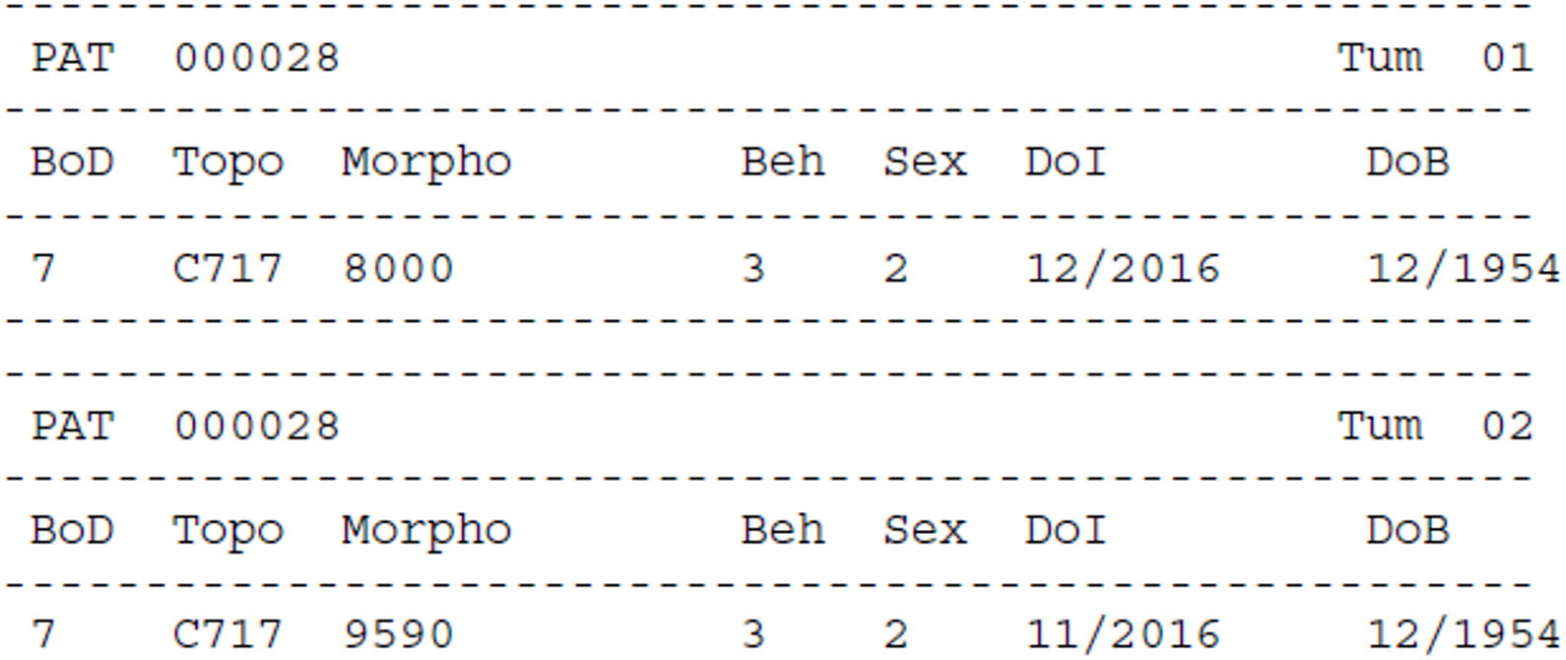
Warnings for multiple primary tumours. In this example of multivariate check, the software gives warning for multiple primary tumours because the two records are reporting the same tumour, and only one should be considered for the calculation of cancer burden indicators.

**Figures 4**–**7** show some examples of errors and warnings given by the JRC-ENCR QCS. Error codes start with an E and warning codes start with a W. The following are examples of a univariate check and of some multivariate checks within the same record. The last example regards a check between different records of the same patient.

For each error and warning the Patient (*Pat*) and Tumour (*Tum*) identifiers are reported. Some essential information on the cancer case is also included: *BoD* (Basis of Diagnosis), *Topo* (Topography), *Morpho* (Morphology), *Beh* (Behaviour), *Sex*, *DoI* (Date of Incidence) and *DoB* (Date of Birth). Finally, the variable(s) that triggered the warning/error are reported (*Var_Name*) with the respective value(s) (*Var_Value*) and resulting code (*Error_Code*).

**Figure 8:**
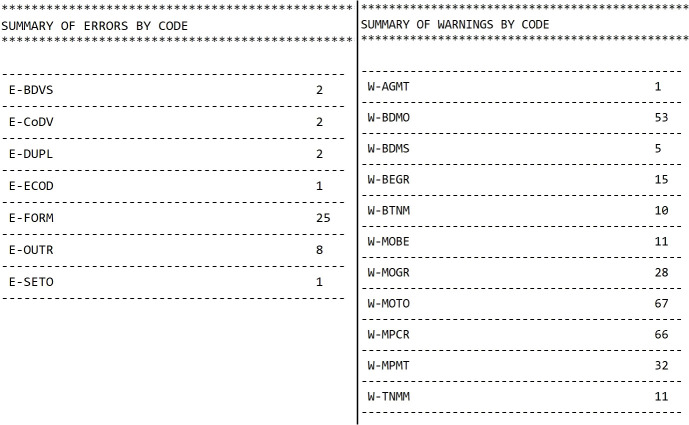
Example of a summary table of errors and warnings provided by the JRC-ENCR QCS.

In addition, the JRC-ENCR QCS is reporting a summary table with type of warnings and errors, and the number of records for each one, to give a general overview of data quality to the user, and help in setting the priorities for reviewing the data (**Figure 8**).

### 3.4 Use of the JRC-ENCR QCS: downloads and trainings

During the period August 2022 - January 2023 there were overall 139 unique downloads of the software. Thirty-two users were from Spain, 28 from Italy, 11 from Germany, 10 from France, 9 from Poland, while the remaining were from about 20 additional countries.

The JRC used the JRC-ENCR QCS for validating the data submitted by the European PBCRs in the 2015 data call for the calculation of incidence and mortality indicators in ECIS. A total of more than 35 million cases from around 150 PBCRs based in 35 countries were processed.

Several training sessions were organised by JRC in collaboration with the ENCR to familiarise PBCRs staff with the software, with around 300 participants trained European-wide.

## 4 Discussion

The JRC-ENCR QCS is a Java standalone desktop application, under development since 2015, created to support PBCRs in their data validation processes.

The software is freely downloadable from the ENCR website (19), allowing the user to work locally and to share data at a later stage in an anonymised/pseudonymised format for European projects. This feature is particularly relevant with respect to compliance to the data protection rules detailed in the GDPR (13).

Since the first release in 2015, the software has been incorporating the updated European and International recommendations and classifications, such as the ICD-O-3.2 (22), the morphology grouping table for the purpose of defining multiple tumours based on the ICD-O-3.2 (19), TNM 8^th^ revision (16) and the ENCR recommendations (23, 24).

Checks related to stage at diagnosis have been improved in the software since its first release. The latest JRC-ENCR QCS version introduced the consistency check of TNM and stage values.

Until the development of the JRC-ENCR QCS, the majority of European PBCRs used *IARCcrgTools* for data validation, a tool developed by the International Agency for Research on Cancer (IARC) (25). Some of the checks performed by the JRC-ENCR QCS related to the core variables are similar to the ones implemented by the *IARCcrgTools*. Therefore, it is possible for non-European PBCRs to use the JRC-ENCR QCS in their data validation process.

The main differences between the two software are the checks related to the TNM staging system, which are implemented in the JRC-ENCR QCS (26). This is a major strength of the software, given the increased number of PBCRs collecting stage, and therefore the necessity to validate this key information. Additionally, the JRC-ENCR QCS also differs from IARC’s software as for checking topography and morphology combinations. While *IARCcrgTools* follows the groups of families included in Appendix 1 of the “Check and Conversion Programs for Cancer Registries” manual (25), the JRC-ENCR QCS is developed around table 8 of the JRC Technical Report (11) which considers each ICD-O-3 topography and morphology code.

In addition, the checks implemented in the JRC-ENCR QCS follow the Call for Data Protocols for European PBCRs variables, formats and allowed values (5, 13). Checks within records (within a single variable or between variables) and between records (duplicate and multiple primary tumours) are performed according to the JRC Technical reports on data quality checks (9–11). The fact that the JRC-ENCR QCS follows the specifications of the Call for Data Protocols for European PBCRs contributes to the improvement of the harmonisation and comparability of data across Europe.

The file format, the number and type variables and the specific ENCR Recommendations should be taken into account by non-European PBCRs using the JRC-ENCR QCS.

To facilitate the submission to the JRC and improve the quality of PBCRs data, the ENCR and the JRC recommend checking beforehand the format of required files and data internal consistency with the JRC-ENCR QCS.

To support the use of the software, the JRC organised in collaboration with the ENCR and other stakeholders several training sessions, with an overall participation of around 300 PBCRs staff so far. In addition, the JRC developed the JRC CSV Data layout converter, a tool to further facilitate the use of the software by PBCRs.

The JRC-ENCR QCS is used by the 15 Swiss PBCRs for the annual submission to the National Agency for Cancer Registration (NACR) (27). The findings are reported back to Swiss PBCRs for correction/verification, and once resolved the data is integrated into the Swiss National Cancer Dataset. Moreover, the software has been used to check data in several studies from European PBCRs and other research institutes (28–35).

The feedback from the JRC-ENCR QCS users has been essential for improving and adapting the software to European PBCRs needs. This feedback allowed to refine the software algorithms in each release and to increase the precision, the completeness and clarity of the software reports.

The flexibility and the performance of the JRC-ENCR QCS has been enhanced over time. In the latest release an innovative approach was introduced, moving all protocol data from the source code to configuration files. This new approach has made it possible to update the logic of majority of checks without the need to release a new software version, but simply updating the configuration files. If needed, user can customise different tables, modifying or introducing specific values.

## Data availability statement

The original contributions presented in the study are included in the article. The JRC-ENCR QCS is accessible at: https://encr.eu/sites/default/files/QCS/jrc-qcs-2.0.zip. The user compendium of the JRC-ENCR QCS is accessible at: https://encr.eu/sites/default/files/QCS/JRC127031_jrc127031_jrc-encr_qcs_user_compendium_v2_0_20220929.pdf. The executables of the JRC-ENCR QCS will be published at: https://code.europa.eu/ecis/jrc-encr-qcs-binaries. Further inquiries can be directed to the corresponding authors.

## Author contributions

The first draft of the manuscript was written by FG and CM. EB supervised the IT process. All authors contributed to the article and approved the submitted version.
